# Genome-wide characterization and comparative phylogenomics of three *Salmonella* Abortusequi strains isolated from equine abortions in Kazakhstan

**DOI:** 10.14202/vetworld.2025.1571-1580

**Published:** 2025-06-16

**Authors:** Temirlan Bakishev, Asylulan Amirgazin, Marat Kuibagarov, Alexander Shevtsov, Zhanar Bakisheva, Gulzhan Yessembekova, Alma Kairzhanova, Ablaikhan Kadyrov, Kui Guo, Xiaojun Wang, Sarsenbay Abdrakhmanov, Sergey Borovikov

**Affiliations:** 1 Department of Veterinary Sanitation, Faculty of Veterinary and Animal Husbandry Technology, S. Seifullin Kazakh Agrotechnical Research University, Astana, Kazakhstan; 2 National Center for Biotechnology, Astana, Kazakhstan; 3 Harbin Veterinary Research Institute, The Chinese Academy of Agricultural Sciences, Harbin, China

**Keywords:** Antimicrobial resistance, equine abortion, Kazakhstan, pathogenicity islands, phylogenomics, *Salmonella* Abortusequi, whole-genome sequencing

## Abstract

**Background and Aim:**

*Salmonella* Abortusequi is a significant etiological agent of equine abortions, yet limited genomic data exist, particularly in Central Asia. This study aimed to perform the first genome-wide characterization and phylogenetic analysis of three *S*. Abortusequi strains isolated from equine abortions in different regions of Kazakhstan.

**Materials and Methods:**

Whole-genome sequencing was conducted on three isolates using the Illumina MiSeq platform. Genomic assemblies were annotated using SPAdes and Prokka, while phenotypic traits were predicted through BioNumerics. Antimicrobial resistance genes, virulence factors, and prophage elements were identified using established databases. Phylogenetic relationships were examined through whole-genome single-nucleotide polymorphism (wgSNP) analysis against a global panel of *S*. Abortusequi and related serovars.

**Results:**

All isolates displayed high genomic similarity and were classified as *Salmonella enterica* subsp. enterica serovar Abortusequi with an antigenic profile of 4:a:e,n,x. Twelve Salmonella pathogenicity islands and three prophages were identified, with ST64B present in all isolates. The ac(6’)-Iaa gene, which confers resistance to aminoglycosides, was detected in all strains. Each genome encoded 101–109 virulence factors, with 94 conserved across isolates. wgSNP analysis confirmed close phylogenetic clustering of the Kazakh strains, with regional variation between northern and southern isolates. Prophage-associated virulence elements, particularly virulence factor protein (SseK), were also documented.

**Conclusion:**

This study reveals the genetic uniformity and virulence potential of *S*. Abortusequi strains circulating in Kazakhstan. The presence of conserved resistance and virulence genes, including prophage-encoded elements, underscores the pathogenic risk posed by these isolates. These findings contribute valuable genomic data for surveillance, diagnosis, and control of salmonellosis in equine populations. Despite the limited sample size, the study establishes a foundation for future genomic epidemiological studies and targeted disease mitigation strategies.

## INTRODUCTION

Salmonella is among the most prevalent Gram-negative bacterial pathogens affecting both animals and humans. In equine production systems, salmonellosis poses a significant health challenge [1]. Equine abortus salmonellosis, a bacterial infection, typically leads to abortion between the 6^th^ and 11^th^ months of gestation. Affected mares often present with lethargy, anorexia, and a prolonged yellowish-brown vaginal discharge persisting for 2–3 weeks. However, abortion due to *Salmonella enterica* serovar Abortusequi can only be provisionally diagnosed based on clinical signs and pathological findings in aborted fetuses. A definitive diagnosis requires bacteriological isolation of the pathogen from clinical samples [2] and confirmation through advanced molecular diagnostic techniques [3–7].

Salmonella-induced abortion in mares has been reported in numerous countries and is associated with considerable economic losses in the equine industry [8–10]. In Kazakhstan, where the horse population is substantial, such infections are particularly detrimental [11, 12]. Although *Salmonella* strains are frequently isolated from mares undergoing abortion and from aborted fetuses, identification of these isolates in Kazakhstan has typically been limited to serotyping. Notably, Daugalieva *et al*. [13] identified single-nucleotide polymorphisms (SNPs) in the *rpsL* gene fragments of vaccine and control strains of *S*. Abortusequi through molecular genetic typing.

Contemporary molecular techniques now enable reliable differentiation of microbial serogroups through genotyping approaches [14]. For instance, genotypic profiling has been employed to characterize *S*. Abortusequi strains isolated from diarrheic horses in Argentina [15]. In Japan, a study of 20 *S*. Abortusequi isolates revealed two distinct pulsed-field gel electrophoresis profiles with a similarity index of 90.9%. However, when these Japanese isolates were compared with five *S*. Abortusequi strains from other countries, the similarity indices ranged from 29.8% to 37.5%, highlighting considerable genetic diversity among global strains [16, 17].

Despite the global recognition of *S. enterica* serovar Abortusequi as a causative agent of equine abortion, there remains a substantial paucity of genomic data, particularly from Central Asian regions such as Kazakhstan, where equine production plays a vital economic and cultural role. Most diagnostic efforts in the region have relied on conventional serotyping methods, which are limited in their capacity to elucidate strain diversity, evolutionary relationships, and virulence potential. Although isolated efforts have explored SNP-level differences in individual genes, no comprehensive genomic investigations have been undertaken to characterize S. Abortusequi strains at a whole-genome scale. Furthermore, there is a lack of integrated analyses combining antimicrobial resistance profiling, virulence gene mapping, and phylogenetic comparisons with globally circulating strains. This gap hinders the development of effective surveillance, diagnostic, and preventive strategies tailored to the regional epidemiology of equine salmonellosis.

The present study aims to conduct the first genome-wide characterization and comparative phylogenomic analysis of S. Abortusequi strains associated with equine abortions in Kazakhstan. Specifically, the study employs high-throughput whole-genome sequencing (WGS) to investigate the genomic architecture of three isolates obtained from geographically distinct regions. The objectives include identifying antimicrobial resistance genes, virulence-associated determinants, and prophage elements, as well as elucidating the phylogenetic relationships of local strains in comparison with previously characterized global isolates. By bridging the present knowledge gap, this study seeks to provide foundational genomic insights that will support more accurate epidemiological surveillance, targeted diagnostic development, and informed intervention strategies to mitigate the impact of S. Abortusequi in equine populations.

## MATERIALS AND METHODS

### Ethical approval

All animal-related procedures were conducted with the approval of the Local Ethical Committee of S. Seifullin Kazakh Agrotechnical Research University, Astana, Kazakhstan (Protocol No. 3, dated 08 November 2023). Sample collection followed the veterinary and sanitary guidelines outlined in the “Rules for Sampling of Transported Objects and Biological Material” (Order No. 7-1/393, dated 30 April 2015). Tissue samples were collected post-mortem from parenchymatous organs of aborted fetuses, specifically the liver, lymph nodes, spleen, lungs, kidneys, as well as from stomach contents, jejunum, colon, and cecum.

### Study period and location

This study was conducted from November 2023 to November 2024 in three regions of Kazakhstan: Karaganda, Akmola, and Zhambyl. One isolate of *S*. Abortusequi was obtained from each region for WGS and comparative genomic analysis.

### Sample collection and bacterial isolation

Biological samples were collected following reports of equine abortions from horse breeding farms operating under a herd housing system. Abortions were observed as isolated cases in some regions and as clusters in others (notably Karaganda).

Tissue samples were collected post-mortem from parenchymatous organs of aborted fetuses – specifically the liver, lymph nodes, spleen, lungs, kidneys – as well as from stomach contents, jejunum, colon, and cecum. In addition, vaginal mucus was collected from the corresponding aborted mares. All samples were stored in commercial Amies Transport Medium with charcoal (Condalab, Spain, #1535). Data on previous abortion cases in the herd, the physiological status of mares, and gross pathological changes in the aborted fetuses were also recorded.

Samples were inoculated on selective Endo agar or bismuth sulfite agar and incubated at 37°C for 18–24 h (or up to 48 h for bismuth sulfite agar). Colonies exhibiting typical *Salmonella* morphology were subcultured for species-level confirmation and genomic analysis.

### Species identification

Species identification was performed through 16S ribosomal ribonucleic acid (rRNA) gene sequencing and matrix-assisted laser desorption/ionization time-of-flight mass spectrometry (MALDI-TOF MS). Amplification and sequencing of the 16S rRNA fragment (~800 bp) were performed using established protocols [18], and species identification was confirmed by BLAST comparison against the National Center for Biotechnology Information (NCBI) nucleotide database (identity threshold >98%).

MALDI-TOF MS was conducted using the Microflex LT Biotyper 4.0 system (Bruker Daltonics, Germany). Spectra were obtained using 40 laser pulses at 60 Hz over a mass range of 2000–20,000 Da and analyzed with Biotyper v4.0 software. Identification scores >2.000 were considered reliable. Only isolates confirmed as *Salmonella* spp. were selected for next-generation sequencing. No technical replicates or resequencing were performed.

### Genomic DNA extraction and WGS

Genomic DNA was extracted using the QIAamp DNA Mini Kit following the manufacturer’s protocol. DNA concentration was quantified using a Qubit double-stranded DNA HS Assay Kit (Thermo Fisher Scientific, Waltham, USA). Sequencing libraries were prepared with the Nextera XT DNA Library Preparation Kit (Illumina, San Diego, USA). The quality and size distribution of the pooled libraries were assessed by agarose gel electrophoresis (1.5% in 1× Tris-acetate-EDTA buffer).

Sequencing was performed on an Illumina MiSeq platform using the MiSeq Reagent Kit v3 600 cycles (catalog number MS-102-3003, Illumina). Standard ultraviolet sterilization protocols were implemented to prevent laboratory contamination. No control strains or technical duplicates were included.

### Genome assembly and annotation

Raw sequence data underwent quality control using FastQC v0.11.7 [19] (https://github.com/s-andrews/FastQC) and MultiQC v1.28 [20] (https://github.com/MultiQC/MultiQC). Trimming was performed using Seqtk v1.3 (https://github.com/lh3/seqtk) with parameters “-b 20 -e 3” and Sickle v1.33 (https://github.com/najoshi/sickle) with parameters “-t sanger -q 30 -g” [21]. Genome assembly was completed using SPAdes v3.15.2 [22] (https://github.com/ablab/spades) with “-k 127 --careful” parameters.

Assembly quality and genome annotation were assessed using Quast v5.0.2 [23] (https://github.com/ablab/quast) and Prokka v1.14.5 [24] (https://github.com/tseemann/prokka), respectively. Phenotypic trait predictions and genomic typing were conducted using the *Salmonella* genotyping plugin in BioNumerics v8.1 (Applied Maths, BioMérieux, Sint-Martens-Latem, Belgium). Resistance and virulence genes were identified using default settings, with a minimum sequence identity threshold of 95% and coverage threshold of 95%, employing the “Combine Fragments” option to reconstruct full-length genes from fragmented contigs.

Prophage identification was performed by querying the *Salmonella* Full Phage KB database v2021.04.12 through BioNumerics v8.1 (Applied Maths, BioMérieux, Sint-Martens-Latem, Belgium) using identity and coverage thresholds of 80% and 40%, respectively. A comprehensive list of software, input/output formats, and configuration parameters is provided in Supplementary Table S1.

### Phylogenetic analysis

Whole-genome SNP (wgSNP) analysis was performed using BioNumerics v8.1 to assess phylogenetic relationships among serovars in Group O:4 (B) [25]. Assemblies of three study strains and reference genomes from NCBI (accessed 12 September 2023) were included. Assemblies were converted into synthetic reads (50 bp length, 10× coverage) for SNP calling through read mapping.

The reference genome used was *S. enterica* serovar Typhimurium ST4/74 (GenBank accession NC_016857). Mapping parameters followed established methods [26]. Phylogenetic clustering was conducted using the unweighted pair group method with arithmetic mean, and minimum spanning trees were generated based on pairwise SNP differences [27].

## RESULTS

### Strain isolation, species identification, and WGS

Between 2021 and 2023, three cases of equine abortion were investigated across three distinct regions of Kazakhstan. Post-mortem examinations of aborted fetuses revealed consistent pathological features, including subcutaneous edema, pulmonary edema, subpleural hemorrhage, and general organ friability. Selective culture of fetal tissues yielded bacterial colonies with morphologies characteristic of *Salmonella* spp.

Species-level identification of the three isolates was confirmed through two independent methods: MALDI-TOF MS using the Biotyper OC Version 4.0 (Build 11), and sequencing of an approximately 800 bp fragment of the 16S rRNA gene.

WGS was subsequently performed. The number of reads per sample ranged from 1,412,918 to 1,463,230, with an average read length of 300 bp. Genome sizes ranged from 4,698,446 to 4,801,507 base pairs. The number of contigs varied between 31 and 107, with N50 values ranging from 116,706 to 406,610 bp. The sequencing depth ranged from 33× to 52×, indicating high-quality genome assemblies suitable for downstream comparative analyses.

**Phenotypic trait prediction and virulence profiling**

Phenotypic trait prediction was carried out using the genome assemblies of the three newly sequenced *S*. Abortusequi strains, in conjunction with three reference genomes available in NCBI (GenBank accessions: GCA_010749735, GCA_010677545, GCA_007751115). Using the *Salmonella* functional genotyping plugin in BioNumerics v8.1, all six strains were assigned an identical antigenic profile of 4:a:e,n,x.

All isolates harbored the ac(6’)-Iaa gene, encoding an aminoglycoside acetyltransferase enzyme that inactivates antibiotics such as tobramycin, kanamycin, and amikacin by acetylation at the 6′-position. This gene confers resistance to aminoglycosides and poses a therapeutic concern.

Twelve Salmonella pathogenicity islands (SPIs) were identified across the genomes: SPI-1 through SPI-6, SPI-9, SPI-11, SPI-14, and CS54. Four SPIs were fully intact across all genomes, while the remaining eight exhibited partial coverage (59%–95% gene presence). Between 101 and 109 virulence genes were detected per genome, with 94 genes conserved among all three strains (Supplementary Tables S2 and S3).

In addition, prophage analysis revealed that each genome contained up to two out of three detected bacteriophages. *Salmonella* phage ST64B was present in all isolates, while SEN34 was found in four of the six genomes analyzed. Only one isolate (1-S21R-48) carried the SSU5 phage (Table 1).

**Table 1:** The number of encoded virulence factors, prophages, and serotypes of the compared strains.

Strain	Virulence islands												Phage {Coverage (%)/Identity (%)}	Serotype		
	
	SPI-1	SPI-2	SPI-3	SPI-4	SPI-5	SPI-6	SPI-9	SPI-11	SPI-12	SPI-13	SPI-14	CS54 island		O antigen	H1 antigen	H2 antigen
*Salmonella*-2E	41/41	42/44	10/17	10/11	7/8	10/15	4/4	7/9	7/7	3/3	2/2	6/8	ST64B (62/97)	4	a	e, n, x
1-S21R-48	41/41	43/44	10/17	10/11	7/8	10/15	4/4	7/9	7/7	3/3	2/2	6/8	ST64B (56/97); SSU5 (47/97)	4	a	e, n, x
LPG-235	41/41	42/44	10/17	10/11	7/8	10/15	4/4	7/9	7/7	3/3	2/2	6/8	ST64B (60/97); SEN34 (40/99)	4	a	e, n, x
Abortusequi_CFSAN022626	41/41	43/44	10/17	10/11	7/8	10/15	4/4	7/9	7/7	3/3	2/2	6/8	ST64B (60/97); SEN34 (42/99)	4	a	e, n, x
Abortusequi_CFSAN022625	41/41	43/44	10/17	9/11	7/8	10/15	4/4	7/9	7/7	3/3	2/2	6/8	ST64B (61/97); SEN34 (42/99)	4	a	e, n, x
Abortusequi_M264	41/41	43/44	10/17	9/11	7/8	10/15	4/4	7/9	6/7	3/3	2/2	6/8	ST64B (64/97); SEN34 (41/99)	4	a	e, n, x

SPI: *Salmonella* pathogenicity islands

### Phylogenetic analysis

To assess the genetic diversity and evolutionary relationships of the *Salmonella* strains, a wgSNP analysis was performed. This analysis incorporated 75 complete *Salmonella* genomes, including the three newly sequenced strains and three reference strains of *S*. Abortusequi (GCA_010749735, GCA_010677545, GCA_007751115) (Supplementary Table S4).

**Figure 1:**
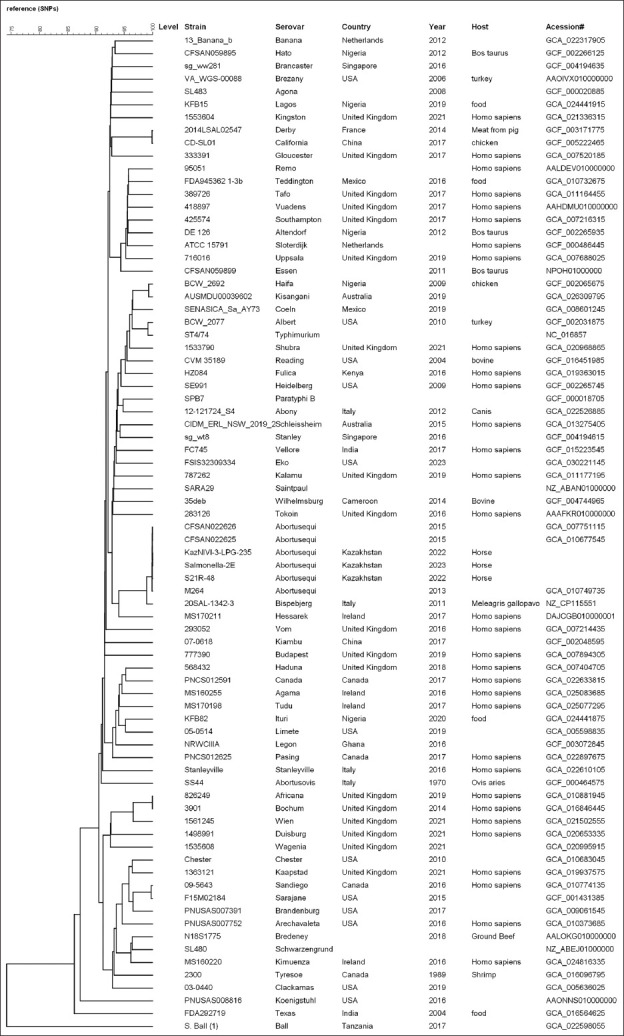
Phylogenetic tree illustrating the genetic evolution of *Salmonella* strains based on whole-genome sequencing.

**Figure 2:**
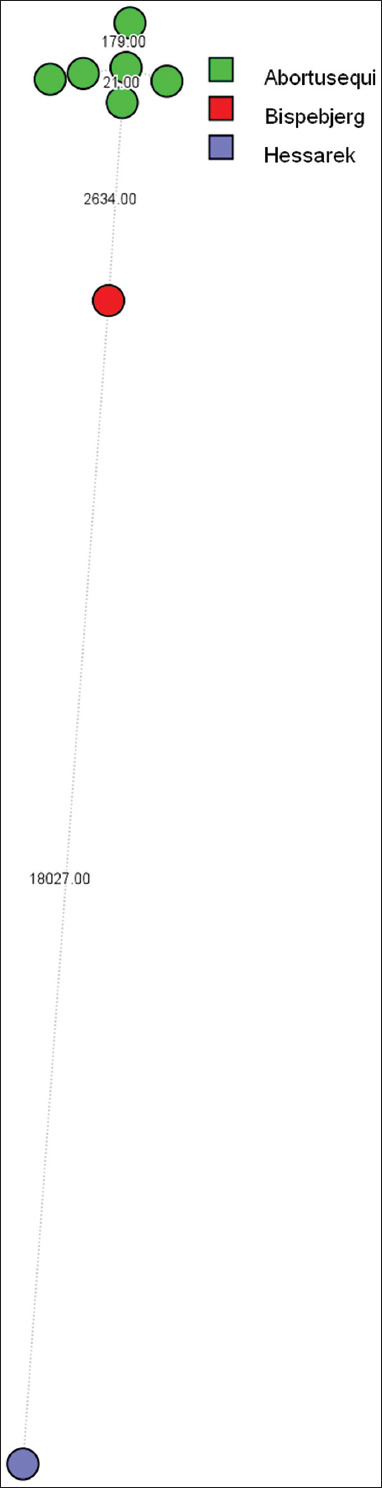
Analysis of the distinction in whole-genome single-nucleotide polymorphisms between *Salmonella* Abortusequi and other *Salmonella* strains.

The isolates from this study clustered within the same phylogenetic lineage as *S. enterica* subsp. *enterica* serovar Abortusequi. Interestingly, this cluster also included strains of serovar Bispebjerg (20SAL-1342-3) and Hessarek (MS170211), suggesting a close evolutionary relationship (Figure 1). The *S*. Abortusequi isolates exhibited over 2600 SNP differences compared to *Salmonella*. Bispebjerg (Figure 2).

**Figure 3:**
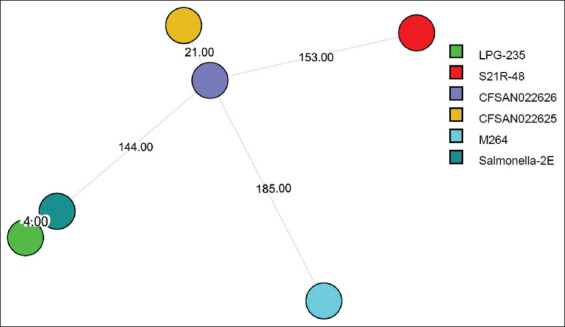
Differential analysis of whole-genome single nucleotide polymorphisms in *Salmonella* Abortusequi isolated from various regions of Kazakhstan.

Within the Kazakhstan isolates, the LPG-235 and Salmonella-2E strains from the southern region differed by only four SNPs, indicating high clonal similarity. In contrast, the S21R-48 strain from the northern region differed by 297 SNPs from Salmonella-2E, demonstrating regional genetic divergence (Figure 3). These findings underscore the utility of wgSNP analysis in differentiating local circulating strains and understanding their geographic distribution.

## DISCUSSION

This study aimed to conduct the first genome-wide characterization and comparative analysis of *S. enterica* subsp. *enterica* serovar Abortusequi strains isolated from equine abortions in Kazakhstan, to understand their genetic structure, virulence potential, and antimicrobial resistance profiles.

### Epidemiological background and historical context

*S*. Abortusequi is a pathogenic serovar with a broad host range, including horses, donkeys, mules, and occasionally other animal species. The serovar has a wide geographic distribution and has been implicated in outbreaks across several countries in Asia, South America, and Europe. In some settings, the disease has re-emerged after prolonged periods of absence, indicating potential for environmental persistence or reintroduction.

In Kazakhstan, historical records show that between 1969 and 1974, mass equine abortion events occurred across multiple farms, with over 50% attributed to *Salmonella* spp. The last reported mass abortion event occurred in 1981. Disease control is complicated by the fact that breeding horses are often kept on remote pastures, making timely detection of abortion cases and sample collection challenging. Despite the known presence of *S*. Abortusequi in the region, prior studies have been limited to phenotypic characterization, with no targeted investigations of the serovar’s antimicrobial resistance or genomic traits [11]. Although a domestic vaccine is available for voluntary use in Kazakhstan, vaccination coverage is inconsistent, particularly for horses raised on private farms.

### Global knowledge and genomic gaps

While extensive genomic studies have been conducted on various *S. enterica* serovars – including Typhi [28], Infantis [29], Gallinarum [30], Telelkebir [31], Dublin [32], Typhimurium [33], and Choleraesui*s* [34] – only one whole-genome analysis of S. Abortusequi has been published to date [35]. That study described a genetically distinct isolate from a donkey in India, which differed by 861 SNPs from known reference strains. The lack of comparative genomic data on *S*. Abortusequi strains from other regions, including Kazakhstan, has limited our understanding of this serovar’s population structure and evolutionary dynamics.

### Genomic homogeneity and surveillance implications

The present analysis of three *S*. Abortusequi strains from Kazakhstan revealed a high degree of genomic similarity, consistent with previous reports from Argentina, Japan, Mongolia, and Croatia [9, 16, 36]. This homogeneity suggests a stable genetic lineage and potentially a common ancestral origin among the strains. These findings underscore the value of genomic epidemiology in tracing transmission dynamics and identifying clonal expansions. WGS can facilitate source attribution during outbreaks and improve the design of molecular diagnostics and surveillance tools tailored to regional epidemiological contexts.

### Antimicrobial resistance determinants

All isolates harbored the ac(6’)-Iaa gene, a member of the aminoglycoside N-acetyltransferase [AAC(6’)] family. This gene mediates resistance to tobramycin, kanamycin, and amikacin through acetylation at the 6′-amine position, although it has limited activity against gentamicin [37]. The detection of this resistance gene in all sequenced strains raises concerns regarding treatment efficacy and highlights the need for routine antimicrobial susceptibility testing in clinical and field settings.

### Virulence gene repertoire and pathogenic potential

Each of the sequenced strains contained a broad array of virulence determinants, with 12 SPIs identified across all genomes. These SPIs encode genes associated with various pathogenic mechanisms, including epithelial invasion, immune evasion, intracellular survival, and toxin production. Between 101 and 109 virulence genes were detected per genome, with 94 shared among all isolates. The conserved virulence profile further indicates a core pathogenic potential that is well adapted to equine hosts.

### Prophage diversity and functional relevance

Prophages represent key vehicles for horizontal gene transfer and contribute significantly to bacterial virulence and evolution [38]. Among the Kazakhstan isolates, the ST64B prophage was consistently detected, with coverage values ranging from 56% to 64% and nucleotide identity of 97%. Originally identified in epidemic strains of *Salmonella* Typhimurium, ST64B is considered a mosaic prophage, comprising genomic segments from non-Salmonella origins [39, 40]. This phage was also previously reported in over 50% of *S*. Typhimurium and *Salmonella* Enteritidis isolates [41].

Of particular interest is the presence of the SseK virulence factor within the ST64B prophage. SseK is a glycosyltransferase that modifies host proteins, including tumor necrosis factor receptor 1 (TNFR1)-associated death domain protein (TRADD) and tubulin-binding cofactor B (TBCB), by arginine glycosylation, thereby disrupting apoptotic signaling and microtubule organization [42]. Such molecular interference supports bacterial survival and host immune evasion.

Additional prophages identified include SSU5**,** a plasmid-associated phage detected in only one isolate (1-S21R-48). SSU5 has been associated with resistance genes against third-generation cephalosporins in other Enterobacteriaceae [43]. SEN34, a lambda-like temperate phage, was detected at approximately 40% coverage in four of the six genomes. The presence of these diverse phages suggests ongoing genomic plasticity and adaptive evolution within local *S*. Abortusequi populations [44].

### Evolutionary dynamics and surveillance recommendations

The incorporation of multiple prophage elements, including those encoding virulence and resistance factors, reveals the complex evolutionary trajectory of *S*. Abortusequi. These elements enhance the pathogen’s ability to adapt to environmental pressures, evade host defenses, and persist within animal reservoirs. Functional validation of these genomic elements is warranted to better understand their phenotypic impacts.

This study also highlights the urgent need for sustained genomic surveillance of *S*. Abortusequi in Kazakhstan. Molecular characterization of a larger number of isolates over broader temporal and spatial scales is essential to capture the full diversity of circulating strains and to inform outbreak response strategies.

## CONCLUSION

This study presents the first genome-wide characterization and phylogenomic analysis of *S*. enterica subsp. enterica serovar Abortusequi strains isolated from equine abortion cases in Kazakhstan. WGS revealed that all three isolates exhibited high genetic similarity, sharing an antigenic profile of 4:a:e,n,x and harboring between 101 and 109 virulence genes, including conserved pathogenicity islands and the aminoglycoside resistance gene *aac(6’)-Iaa*. All strains carried the ST64B prophage, with some also harboring SEN34 and SSU5, highlighting the contribution of prophage elements to genomic plasticity and virulence evolution.

A key strength of this study lies in its integrative use of WGS, functional genotyping, and wgSNP-based phylogenetics to offer a detailed genomic portrait of *S*. Abortusequi in a previously uncharacterized region. The combination of high-resolution molecular typing and resistance gene profiling enhances the present understanding of the strain’s pathogenic potential and evolutionary lineage.

The findings have direct implications for the surveillance, diagnosis, and control of equine salmonellosis. The identification of conserved virulence and resistance determinants provides a molecular basis for the development of region-specific diagnostic assays and supports evidence-based antimicrobial stewardship in equine veterinary practice. Furthermore, wgSNP differentiation between northern and southern strains enables more accurate tracing of outbreak sources and transmission routes.

The study is limited by its small sample size, with only three isolates analyzed. This restricts the generalizability of the findings and may not capture the full genomic diversity of *S*. Abortusequi circulating in Kazakhstan or other host species. In addition, functional assays to validate the expression and phenotypic impact of resistance and virulence genes were not performed.

Future research should involve larger-scale surveillance incorporating multiple geographic locations, time points, and host species. Longitudinal genomic monitoring, coupled with phenotypic antimicrobial susceptibility testing and host–pathogen interaction studies, will be critical for elucidating the dynamics of *S*. Abortusequi evolution and persistence. Studies on vaccine efficacy and the role of prophage-mediated gene transfer in virulence acquisition are also warranted.

In conclusion, this study provides foundational genomic data on *S*. Abortusequi in Kazakhstan and underscores the value of WGS in veterinary pathogen surveillance. The evidence of genomic conservation and the presence of key resistance and virulence determinants point to the importance of sustained genomic epidemiology efforts. These insights will inform the design of diagnostic tools, guide antimicrobial therapy, and support targeted interventions to mitigate the burden of equine salmonellosis in endemic regions.

## DATA AVAILABILITY

All data from this project are publicly available. NCBI Bioproject Accession: PRJNA1258605.

## AUTHORS’ CONTRIBUTIONS

TB: Design and management of the study. AA: NGS sequencing of *Salmonella* isolates. MK: Bacteriological studies and species identification of bacterial cultures. AS: Analysis of sequencing results. ZB and AK: Sampling of biological material. GY: Mass spectrometry of intact cells. AbK: Sequencing of 16S rRNA gene of *Salmonella* isolates. KG and SA: Manuscript editing. XW: Data analysis and translation of the manuscript into English. SB: Data analysis and manuscript writing. All authors have read and approved the final manuscript.

## References

[1] Grandolfo E, Parisi A, Ricci A, Lorusso E, De Siena R, Trotta A, Buonavoglia D, Martella V, Corrente M (2018). High mortality in foals associated with *Salmonella enterica subsp. Enterica* abortusequi infection in Italy. J. Vet. Diagn. Invest.

[2] Zamil S, Ferdous J, Zannat M.M, Biswas P.K, Gibson J.S, Henning J, Hoque M.A, Barua H (2021). Isolation and antimicrobial resistance of motile *Salmonella enterica* from the poultry hatchery environment. Vet. Res. Commun.

[3] Burgess B.A, Noyes N.R, Bolte D.S, Hyatt D.R, Van Metre D.C, Morley P.S (2015). Rapid *Salmonella* detection in experimentally inoculated equine faecal and veterinary hospital environmental samples using commercially available lateral flow immunoassays. Equine Vet. J.

[4] Aribam S.D, Nakayama M, Ichimura S, Tokuyama K, Hara Y, Ogawa Y, Shimoji Y, Eguchi M (2023). Differentiation of *Salmonella* vaccinated and infected animals by serological detection of antibody to T3SS effector SsaK in an indirect ELISA. J. Microbiol. Methods.

[5] Guo K, Guo W, Liu D, Zhang W, Yang Y, Zhang Z, Li S, Wang J, Chu X, Wang Y, Hu Z, Wang X (2023a). Development and application of a competitive ELISA for the detection of antibodies against *Salmonella Abortusequi* in equids. J. Clin. Microbiol.

[6] Guo K, Zhang Z, Yang Y, Zhang W, Wang J, Li S, Chu X, Guo W, Liu D, Wang Y, Hu Z, Wang X (2023b). Development and application of an iELISA for the detection of antibody against *Salmonella* Abortusequi. Transbound. Emerg. Dis.

[7] Wang J, Guo K, Li S, Liu D, Chu X, Wang Y, Guo W, Du C, Wang X, Hu Z (2023). Development and application of real-time PCR assay for detection of *Salmonella* Abortusequi. J. Clin. Microbiol.

[8] Burgess B.A (2023). *Salmonella spp*. in horses. Vet. Clin. North Am. Equine Pract.

[9] Bustos C.P, Moroni M, Caffer M.I, Ivanissevich A, Herrera M, Moreira A.R, Guida N, Chacana P (2020). Genotypic diversity of *Salmonella ser.* Abortusequi isolates from Argentina. Equine Vet. J.

[10] Wang H, Liu K.J, Sun Y.H, Cui L.Y, Meng X, Jiang G.M, Zhao F.W, Li J.J (2019). Abortion in donkeys associated with *Salmonella* Abortusequi infection. Equine Vet. J.

[11] Sultanov A.A, Musaeva A.K, Egorova N.N, Dosanova A.K (2015). Diagnosis and prevention of S*almonella* abortion in mares. Int. J. Appl. Basic Res.

[12] Borovikov S, Kuibagarov M, Akibekov O, Muranets A (2024). Clinical case of *Salmonella* detected in an aborted mother fetus and its characteristics. Int. J. Vet. Sci.

[13] Daugalieva А.Т, Musayeva А.K, Egorova N.N (2017). Мolecular-genetic typing of rpsl gene fragments of *Salmonella* strains. Nat. Acad Sci. Repub. Kazakhstan. Ser. Biol. Med.

[14] Uzzau S, Hovi M, Stocker B.A (1999). Application of ribotyping and IS200 fingerprinting to distinguish the five *Salmonella* serotype O6,7:c:1,5 groups:*Choleraesuis* sensu stricto, *Choleraesuis* var. Kunzendorf, *Choleraesuis* var. Decatur, Paratyphi C and *Typhimurium*. Epidemiol. Infect.

[15] Bustos C.P, Dominguez J.E, Garda D, Moroni M, Pallarols N.P, Herrera M, Chacana P.A, Mesplet M (2021). Multiresistant and bla_CTX-M-14_-carrying *Salmonella ser. Typhimurium* isolated during a salmonellosis outbreak in an equine hospital in Argentina. J. Equine Vet. Sci.

[16] Akiba M, Uchida I, Nishimori K, Tanaka K, Anzai T, Kuwamoto Y, Wada R, Ohya T, Ito H (2003). Comparison of *Salmonella enterica*
*Serovar* Abortusequi isolates of equine origin by pulsed-field gel electrophoresis and fluorescent amplified-fragment length polymorphism fingerprinting. Vet. Microbiol.

[17] Leon I.M, Lawhon S.D, Norman K.N, Threadgill D.S, Ohta N, Vinasco J, Scott H.M (2018). Serotype diversity and antimicrobial resistance among *Salmonella enterica* isolates from patients at an equine referral hospital. Appl. Environ. Microbiol.

[18] Kuibagarov M, Zhylkibayev A, Kamalova D, Ryskeldina A, Yerzhanova N, Ramankulov Y, Shevtsov A, Angelos J.A (2022). Genetic diversity of pilin from Kazakh isolates of *Moraxella bovoculi*. Adv. Anim. Vet. Sci.

[19] Andrews S (2010). Fast QC:A Quality Control Tool for High-Throughput Sequence Data. Version 0.11. 9.

[20] Ewels P, Magnusson M, Lundin S, Kaller M (2016). MultiQC:Summarize analysis results for multiple tools and samples in a single report. Bioinformatics.

[21] Joshi N.A, Sickle F.J (2011). A Sliding-Window, Adaptive, Quality-Based Trimming Tool for Fastq Files. Version 1. 33.

[22] Bankevich A, Nurk S, Antipov D, Gurevich A.A, Dvorkin M, Kulikov A.S, Lesin V.M, Nikolenko S.I, Pham S, Prjibelski A.D, Pyshkin A.V, Sirotkin A.V, Vyahhi N, Tesler G, Alekseyev M.A, Pevzner P.A (2012). SPAdes:A new genome assembly algorithm and its applications to single-cell sequencing. J. Comput. Biol.

[23] Gurevich A, Saveliev V, Vyahhi N, Tesler G (2013). QUAST:Quality assessment tool for genome assemblies. Bioinformatics.

[24] Seemann T (2014). Prokka:Rapid prokaryotic genome annotation. Bioinformatics.

[25] Grimont P.A, Weill F.X (2007). Antigenic formulae of the *Salmonella* serovars. In:WHO Collaborating Centre for Reference and Research on *Salmonella*.

[26] Shevtsov A, Cloeckaert A, Berdimuratova K, Shevtsova E, Shustov A.V, Amirgazin A, Karibayev T, Kamalova D, Zygmunt M.S, Ramanculov Y, Vergnaud G (2023). *Brucella abortus* in Kazakhstan, population structure and comparison with worldwide genetic diversity. Front. Microbiol.

[27] Roer L, Hendriksen R.S, Leekitcharoenphon P, Lukjancenko O, Kaas R.S, Hasman H, Aarestrup F.M (2016). Is the evolution of *Salmonella enterica* subsp. *Enterica* linked to restriction-modification systems?*mSystems*.

[28] Wan Makhtar W.R.W, Bharudin I, Samsulrizal N.H, Yusof N.Y (2021). Whole genome sequencing analysis of *Salmonella enterica*
*Serovar* Typhi:History and current approaches. Microorganisms.

[29] Cohen E, Rahav G, Gal-Mor O (2020). Genome sequence of an emerging *Salmonella enterica*
*Serovar* Infantis and genomic comparison with other *S.* Infantis strains. Genome Biol. Evol.

[30] Vaid R.K, Thakur Z, Anand T, Kumar S, Tripathi B.N (2021). Comparative genome analysis of *Salmonella enterica*
*Serovar gallinarum* biovars Pullorum and *Gallinarum* decodes strain-specific genes. PLoS One.

[31] Qiu Y.F, Nambiar R.B, Xu X.B, Weng S.T, Pan H, Zheng K.C, Yue M (2021). Global genomic characterization of *Salmonella enterica*
*serovar* Telelkebir. Front. Microbiol.

[32] Campioni F, Vilela F.P, Cao G, Kastanis G, Dos Prazeres Rodrigues D, Costa R.G, Tiba-Casas M.R, Yin L, Allard M, Falcão J.P (2022). Whole genome sequencing analyses revealed that *Salmonella enterica*
*serovar* Dublin strains from Brazil belonged to two predominant clades. Sci. Rep.

[33] McClelland M, Sanderson K.E, Spieth J, Clifton S.W, Latreille P, Courtney L, Porwollik S, Ali J, Dante M, Du F, Hou S, Layman D, Leonard S, Nguyen C, Scott K, Holmes A, Grewal N, Mulvaney E, Ryan E, Sun H, Florea L, Miller W, Stoneking T, Nhan M, Waterston R, Wilson R.K (2001). Complete genome sequence of *Salmonella enterica*
*serovar* Typhimurium LT2. Nature.

[34] Chiu C.H, Tang P, Chu C, Hu S, Bao Q, Yu J, Chou Y.Y, Wang H,S, Lee Y.S (2005). The genome sequence of *Salmonella enterica*
*serovar* Choleraesuis, a highly invasive and resistant zoonotic pathogen. Nucleic Acids Res.

[35] Manikandan R, Rajagunalan S, Malmarugan S, Gupta C (2024). First report on whole genome sequencing and comparative genomics of *Salmonella enterica*
*serovar* Abortusequi isolated from a donkey in India. Sci. Rep.

[36] Niwa H, Hobo S, Kinoshita Y, Muranaka M, Ochi A, Ueno T, Oku K, Hariu K, Katayama Y (2016). Aneurysm of the cranial mesenteric artery as a site of carriage of *Salmonella enterica* subsp. *Enterica serovar* Abortusequi in the horse. J. Vet. Diagn. Invest.

[37] Salipante S.J, Hall B.G (2003). Determining the limits of the evolutionary potential of an antibiotic resistance gene. Mol. Biol. Evol.

[38] Sattar S, Ullah I, Khanum S, Bailie M, Shamsi B, Ahmed I, Shah S.T.A, Javed S, Ghafoor A, Pervaiz A, Sohail F, Shah N.A, Imdad K, Bostan N, Altermann E (2022). Phenotypic characterization and genome analysis of a novel *Salmonella* Typhimurium phage having unique tail fiber genes. Sci. Rep.

[39] Mmolawa P.T, Schmieger H, Heuzenroeder M.W (2003). Bacteriophage ST64B, a genetic mosaic of genes from diverse sources isolated from *Salmonella enterica serovar* Typhimurium DT. 64. J. Bacteriol.

[40] Hiley L, Fang N.X, Micalizzi G.R, Bates J (2014). Distribution of gifsy-3 and variants of ST64B and gifsy-1 prophages amongst *Salmonella enterica serovar* Typhimurium isolates:Evidence that combinations of prophages promote clonality. PLoS One.

[41] Mottawea W, Duceppe M.O, Dupras A.A, Usongo V, Jeukens J, Freschi L, Emond-Rheault J.G, Hamel J, Kukavica-Ibrulj I, Boyle B, Gill A, Burnett E, Franz E, Arya G, Weadge J.T, Gruenheid S, Wiedmann M, Huang H, Daigle F, Moineau S, Bekal S, Levesque R.C, Goodridge L.D, Ogunremi D (2018). *Salmonella enterica* prophage sequence profiles reflect genome diversity and can be used for high discrimination subtyping. Front. Microbiol.

[42] Hasan M.K, Scott N.E, Hays M.P, Hardwidge P.R, El Qaidi S (2023). *Salmonella* T3SS effector SseK1 arginine-glycosylates the two-component response regulator OmpR to alter bile salt resistance. Sci. Rep.

[43] Gilcrease E.B, Casjens S.R (2018). The genome sequence of *Escherichia coli* tailed phage D6 and the diversity of enterobacteriales circular plasmid prophages. Virology.

[44] Mikalova L, Bosak J, Hribkova H, Dedicova D, Benada O, Smarda J, Smajs D (2017). Novel temperate phages of *Salmonella enterica* subsp. Salamae and subsp. Diarizonae and their activity against pathogenic S. *Enterica subsp*. *Enterica* isolates. PLoS One.

